# Motivational and Affective Factors Underlying Consumer Dropout and Transactional Success in eCommerce: An Overview

**DOI:** 10.3389/fpsyg.2020.01546

**Published:** 2020-07-03

**Authors:** Lynne Bell, Rachel McCloy, Laurie Butler, Julia Vogt

**Affiliations:** ^1^School of Psychology and Clinical Language Sciences, University of Reading, Reading, United Kingdom; ^2^Faculty of Science and Engineering, Anglia Ruskin University, Cambridge, United Kingdom

**Keywords:** consumer behavior, online checkout, consumer dropout, shopping cart abandonment, online payment, eCommerce

## Abstract

It is estimated that more than half of all online transactions are abandoned before completion. This paper investigates the psychological factors that influence online shopping behavior, with a view to improving transactional success rates. Through a review of the literature, we identify a range of factors which predict abandonment of online shopping, highlighting affective and motivational dimensions in addition to processing style and characteristics of the consumer, device, and product. We conclude that online purchasing and payment systems that boost consumers’ motivation to buy and prevent or attenuate negative affective states will demonstrate the greatest rates of transactional success. However, with rapid advancement in technology, continued research is needed to fully understand the potential impact on future online purchasing behavior.

## Introduction

It is estimated that more than half of all online transactions are abandoned before completion. So called consumer dropout (also known as shopping cart abandonment) is therefore of great interest to both retailers and scholars. It describes the behavioral outcome of leaving items in an online shopping cart without completing a purchase session (cf. Huang et al., 2018). Estimates of abandonment rates range from 25-75% of transactions (Sheth, 2013; Worldpay, 2016; Baymard Institute, 2019), and data collected from studies of actual online transactions suggest the true value to sit firmly in the upper range of this estimate. For example, a commercial study of online metric data (Sheth, 2013) revealed that 40–50% of potential transactions were abandoned at the first stage of the checkout process where consumers are requested to login or register as a new customer.

The relevance of understanding shopping cart abandonment is also reflected in the rising frequency of online shopping. Online shopping (eCommerce) has increased to the point where more than half of all purchases, made by shoppers who shop both online and offline, are now made online (UPS, 2016). “Millennial,” shoppers born between 1982 and 1993, made 54% of their purchases online. In “Non-Millennials” the figure was slightly lower at 49%. Latest figures for the United States suggest that online commerce now forms a major part of all retail (US Census Bureau, 2019). Indeed, a recent forecast predicts that eCommerce will achieve a global market share of greater than 17% by 2022 (451 Research, 2018). The rise in eCommerce due to the lockdown in several countries during the covid-19 pandemic might further enhance this effect, even after the lockdown measures are finished (WARC, 2020). This strongly suggests that online shopping will become even more popular in the future. In global commercial terms, shopping cart abandonment therefore potentially represents more than $4 trillion in lost sales annually (Business Insider, 2014).

Moreover, online purchasing has been associated with the adoption of mCommerce technology (Wang et al., 2015), where online shopping is performed using mobile devices including phones and tablets. It is posited that this is due to increased convenience and the formation of habitual online shopping behavior, particularly for low cost, repurchase items. Indeed, in the United Kingdom, for example, 31% of all eCommerce traffic is already reported to be carried out on mobile devices (Twenga Solutions, 2015). Therefore, models of shopping cart abandonment need also to consider the device the consumer is using.

The goal of this paper is to shed light on why consumers buy or not when online shopping. We aim to provide an overview of the current state of research in this area and to discuss avenues for future research.

### Method

A literature search of Google Scholar, Science Direct, and Emerald Insight was performed using relevant search terms: (online or internet or web or net or app or eCommerce or mCommerce) and (payment or checkout or transaction or shopping or retail or purchase or “shopping cart” or “shopping basket”) and (behavior or psychology or attitude or “decision making” or abandon or demographic or gender or age). Search dates were from 1995 onward. Additional literature was sourced using citation linking from previously sourced articles.

Based on broad topics identifiable from the review, we have divided the sourced literature into motivational and emotional factors that influence shopping cart abandonment. We then used seminal work from motivation and emotion research to provide definitions of key concepts and processes. Further, we have categorized demographic differences in behavioral response to these factors. Finally, we have identified research findings that cannot be summarized under any of these factors. The discussion addresses additional frameworks and theories to incorporate these findings.

### Framework for the Review

In this paper, we will review previous work pertinent to understanding online buying decisions. Previous models of online shopping have considered important practical aspects of the shopping process such as the sequential processes involved (e.g., Comegys et al., 2006) and the organization and research of products within the shopping cart (e.g., Xu and Huang, 2015). Here we will review motivational and emotional, and cognitive processing factors that are inherent within the shopping process and that play a significant role in shopping cart abandonment. Our review will be guided by the assumption that consumers will buy (1) when they have the motivation to buy, and (2) when this motivation is not inhibited but rather fostered during the online search, evaluation and buying process. To this end, we follow classic models of buying behavior that suggest the buying process starts with the intention to buy (or need recognition, Howard and Sheth, 1969). Importantly, if the motivation to buy is not the reason for a user to visit an online store, we assume that buying is unlikely, as most behaviors reflect people’s current and prioritized goals (Kruglanski et al., 2002; Kukar-Kinney and Close, 2010; Song, 2019). However, and in contrast to the classic model of buying behavior, we assume that the motivation to buy can be installed even when it was not the original motivation, such as when a consumer who only planned to browse is presented with attractive offers (Cialdini, 2001). We also consider the possibility of impulse buying which reflects a bottom-up cause of buying in response to tempting products and is a main cause of buying now (Baumeister, 2002; Vohs and Faber, 2007; Li and Wang, 2015). All these considerations reveal that the motivation to buy can be activated at later stages than originally predicted by classic models of buying behavior (Howard and Sheth, 1969), and does not necessarily need to reflect a conscious, strategic decision (Chartrand and Fitzsimons, 2011).

Further, we consider emotional and affective factors that can inhibit or foster buying. Of course, factors that prevent buying in the store will also affect buying decision in online environments. For instance, concerns regarding the price of an item, over choice (where the customer is overwhelmed by the range of similar products available; Toffler, 1970; Lee and Lee, 2004), or insufficient choice or information are significant factors in shopping cart abandonment (Cho et al., 2006; Park and Kim, 2010). Confidence in the product, brand, or internet purchase process (Hahn and Kim, 2009) is also of importance. Once online, technical problems might simply interrupt an online shopping experience. However, we will focus on affective and emotional factors caused by specific characteristics of the online shopping situation that can enhance the motivation to buy, such as when consumers experience the navigation of a website as fluent or safe, but also those that can undermine or prevent buying. For instance, when security fears are evoked, or the shopping experience is disfluent because of website layout or navigation, consumers will likely abandon it (Cho et al., 2006; Sørebø, 2018; Indiani and Fahik, 2020). Our reasoning is grounded in dominant theories of affect, emotion, and motivation. These theories suggest that negative feelings and emotions signal an individual to be careful (Schwarz and Clore, 1983, 2003), or that an activity is not valuable (Fishbach et al., 2010). Consequently, most negative emotions or moods will cause a tendency to withdraw and stop whatever the person is doing at that moment in order to analyze the situation more carefully (Schwarz and Clore, 2003; Moors, 2016), or cause them to abandon the shopping process entirely (Louro et al., 2007; Fishbach et al., 2010) if negative affect or emotions are not attenuated. In line with our reasoning, hesitation is a key factor in predicting online shopping cart abandonment, particularly at the final payment stage where security fears or fear of regret may lead to reconsideration of the purchase (Cho et al., 2006; Erdil, 2018). In contrast, positive emotions such as trust are likely to promote buying because they are related to a more superficial processing mode, and signal that the situation is safe (Schwarz and Clore, 1983, 2003; Lee and Turban, 2001). In sum, we argue that positive emotions will promote buying whereas negative emotions will reduce the likelihood of buying throughout the online shopping process. Importantly, we discuss how negative mood and emotions can be attenuated when they occur during the online shopping process (cf. Tang and Lin, 2019).

We will also use this framework to integrate studies from other fields that are directly related to online shopping questions and to point the way for future research directions. For instance, we will discuss processing mode (automatic versus strategic) as a moderator of intention to buy. Below we explain the basic components of the framework and review previous findings highlighting how motivational and emotional factors and mode of processing might cause or prevent shopping cart abandonment. As part of this review, we will also investigate how specific characteristics of the consumer, such as their age, gender, and type of device they are using, moderate the link between emotion, motivation, and online shopping cart use.

## Motivational Factors

### Theoretical Background: Goals

A goal can be understood as the cognitive representation, or schema, of a desired end state that a person aims to achieve (Fishbach and Touré-Tillery, 2013). The goal representation, once activated, impacts emotion, behavior, and cognitive processes (Kruglanski et al., 2002). For instance, the activation of the goal to buy a new pair of running shoes might tune cognitive, affective, and behavioral processes toward items related to running, and might thus mean that a buyer visits an online store offering running shoes.

Importantly, only goals that have high value and success expectancy (i.e., seem achievable) to the individual will be prioritized and pursued in each situation (Fishbach and Ferguson, 2007; Huang et al., 2018). Moreover, the importance of a goal will impact likelihood of buying. For instance, if a buyer experiences a price as too high, and thus her ability to pay for an item as unrealistic, they will very likely not buy. Relatedly, if a consumer is motivated to browse but not to buy, buying is unlikely (Kruglanski et al., 2002). It is important to note that perception of value and achievability of a goal such as buying can change quickly and dynamically (e.g., Fishbach and Dhar, 2005; Louro et al., 2007). For instance, learning that an item is scarce boosts value (Cialdini, 2001). Related to this, tempting products can evoke impulse buying (Li and Wang, 2015). However, mental accounting at the checkout may impact achievability (Sondhi, 2017). Further, much research in the last two decades has demonstrated that the activation and pursuit of goals is not limited to strategic processes. Goals can be unconsciously activated (e.g., Chartrand et al., 2008) and even when goals are strategically activated, goal pursuit will be accompanied by the operation of automatic processes (for an overview see Ferguson et al., 2008). For instance, the goal to lose weight might be activated automatically when seeing an advertisement showing a slim model. In the same vein, when having consciously decided to lose weight, automatic processes will accompany goal pursuit, such as the automatic activation of goal-relevant information (e.g., thoughts about healthy food, Fishbach et al., 2003). In what follows, we will describe how varying motivations prevent or facilitate transactional success and how motivation to buy can be boosted or installed.

#### Primary Motivation to Buy Is Needed in Order to Buy

When considering online shopping goals, it is important to understand consumer motivations for placing products in the cart in the first instance. In offline shopping the assumed motivation is purchase intention, however, research suggests that this assumption may not be valid for online shopping. Kukar-Kinney and Close (2010; see also Baymard Institute, 2019; Song, 2019) observed that factors relating to online search, such as consideration and organization of search items, were greater predictors of shopping cart abandonment than failures at the final purchase decision stage, such as cost or security concerns. This confirms that alternative uses of the shopping cart have a strong influence on transaction completion. Similarly, Close and Kukar-Kinney (2010) showed that organizational intent where the cart was used as a wish list, to bookmark items for later, or to narrow items down after further evaluation, was a driver of shopping cart use. In contrast, consumers who browsed for entertainment tended not to use the cart (see also Erdil, 2018). The shopping cart was also not routinely used for information gathering. Therefore, many instances of cart abandonment, or not even using the cart, result from alternative goals where purchase was never intended.

It is important to note that individual differences will affect whether consumers tend to buy or browse. In a study of browser/buyer differences (Lepkowska-White, 2004) self-reported browsers (who used the internet as a search tool but made all subsequent purchases in store) showed much greater concerns over website design, security, customer service, and product quality. Browsers also rated themselves as more price conscious, less time pressured, and less skilled at using the internet compared with online buyers. Further research has identified several distinct groups of browsers, based on their different barriers to online purchasing (Iglesias-Pradas et al., 2013). Four main barriers to online shopping were identified: distrust; lack of resources; low availability of desired products; poor computer literacy. Additional investigation of drivers to incentivize a purchase revealed that lower risk, product availability, availability of low-cost internet access, and easy to use platforms might influence these consumers to buy online. Therefore, addressing these factors through better online design, information, and training may help to attract this section of the consumer base. Specific methods of fear reduction are considered below.

#### Increase Motivation to Buy

In this section, we highlight how motivation to buy can evolve during the shopping process. For instance, incentives could help to change the motivations of consumers who have placed items in their cart for purposes other than immediate purchase. Consumers will adopt a goal to buy now when this goal seems appealing (e.g., buying now allows the saving of money), urgent and/or achievable (Fishbach and Ferguson, 2007). For instance, Close and Kukar-Kinney (2010; see also Huang et al., 2019) found that a desire to take advantage of special offers such as sales, price promotions and free shipping predicts shopping cart use. For those consumers already using the basket as a cost-comparison mechanism, the use of price guarantees can be a powerful incentive to help the consumer stop their search and proceed to buying (McConnell et al., 2000).

What about more general browsers, who are using the basket as a wish list or external memory? The additional incentive of a visible and time-limited reduction in price has the potential to trigger the consumer to change mode from browsers to buyers by providing a justification for their immediate purchase (Shafir et al., 1993). Providing additional incentives as justification may be particularly strong for purchases that are hedonic (for fun) as opposed to utilitarian (necessary) (Okada, 2005). The value and urgency of products can also be increased by emphasizing that availability is scarce. This can be achieved by suggesting that only a few items are left or that an item or price will only be available for a certain time (Worchel et al., 1975; Cialdini, 2001).

In a similar vein, a survey of online clothing retail (Kim and Kim, 2011) found that incentive programs were the biggest predictor of purchase intention in this sector, and high earning women with children were the most frequent consumers. Indeed, female shoppers also show greater satisfaction in online sectors such as fashion retail, where bargain shopping has been likened to a competitive sport amongst a sub-group of women (O’Donnell et al., 2016).

Although offering coupons is shown to act as a positive incentive, the way in which customers are required to enter the information at checkout can impact significantly on purchasing behavior (Oliver and Shor, 2003). In an online shopping experiment, subjects with a promotional code were influenced by the wording used to prompt its entry. Improved ratings of satisfaction and fairness were observed when the term “discount” was used rather than “promotion” or “coupon”; a possible explanation is that “discount” has a concrete semantic meaning that is strongly related to price. However, subjects without a code reported lower ratings of fairness, satisfaction, intention and, importantly, increased non-completion rates, when prompted at the checkout to enter one. In this instance the description of the code was not found to be important.

The value of a goal or activity also reflects social influence in that social norms drive what consumers value (e.g., Melnyk et al., 2010; Hu et al., 2019). Consequently, female consumers are more likely to make online purchases if the product is promoted by word of mouth recommendation, for example through reward schemes for recommending a friend (Garbarino and Strahilevitz, 2004) or by recommendations to consumers based on other consumers’ feedback (Wang et al., 2007; Meyers-Levy and Loken, 2015). Similarly, humanizing a website seems to make it more attractive especially to female shoppers and increases the likelihood of buying. For instance, online avatars seem to enhance an emotional bond with the website and enhance perceptions of human connection (Wang et al., 2007). Relatedly, online shopping enjoyment increases simply by presenting images of models wearing the garments (Hassanein and Head, 2007). Further, personalized online recommendations for products of interest improve women’s online shopping experience (Baier and Stüber, 2010). Recommendations based on other consumers feedback or using avatars to impart the recommendations has the potential to increase the immediate social presence of the site and attract more women to buy (Wang et al., 2007; Meyers-Levy and Loken, 2015).

By offering different options for payments (e.g., delayed payments), payment can be made more *achievable* for certain customers. For example, the availability of a deferred payment option is a strong predictor of online spending in low earners, but not in high earners (Hannah and Lybecker, 2010). Indeed, revenue increases are likely to be associated with the introduction of deferred payment systems (Hannah and Lybecker, 2010). Additionally, consumers might not buy when they do not feel competent to use their device (Huang et al., 2018). Thus, in addition to the perception of being able to pay, that is, having the financial resources to buy a product, the consumer also needs to feel capable to conduct the financial transfer online.

#### Spontaneous Purchases

Buying may sometimes also occur impulsively during the online browsing process. Indeed, Stern (1962) predicted an increase in spontaneous purchasing in self-service environments, facilitated by additional factors including low price, wide availability, mass advertising and prominent store display. Reduced levels of self-control have been posited as a likely predictor of spontaneous purchase (Baumeister, 2002). Self-control lapses are often explained by missing capacity to exert control due to continuously or momentarily impaired ability to engage in self-control (see Fujita, 2011; Kotabe and Hofmann, 2016, for overviews). However, self-control can also be hampered by situational factors, especially when the costs of indulging in temptation are blurred, and rational and slow decision making is prevented (Shefrin and Thaler, 1981; Fishbach and Zhang, 2008). Such research presents impulse buying as a weakness, but there is also some evidence that impulse buying may be viewed as an acceptable or even positive outcome in certain situations (Rook and Fisher, 1995), moderated by normative evaluation of the situational context. Impulse buying norms can therefore be actively promoted within the online shopping environment. With regard to self-control, website design can further facilitate such spontaneous purchases, although the inherent impulsiveness of the consumer is also a mediating factor (Wells et al., 2011). In an online shopping experiment (Dutta et al., 2003), usability, feedback and rehearsal factors were manipulated across several conditions. High usability required only a single stage to complete a purchase whereas low usability required three. Feedback reminded participants of the amount spent in each transaction. Rehearsal required consumers to type in the amount they needed to pay. It was found that low rehearsal combined with high usability promoted the greatest propensity to make spontaneous purchases as measured using an exit survey (see also Korzaan, 2003). Overall, the study outcome provides evidence for one-click purchasing leading to spontaneous purchase.

Similarly, self-control theory assumes that consumers will indulge when the value of the temptation is boosted (e.g., Hofmann et al., 2010). In line with this reasoning, Shen and Khalifa (2012) found that reaffirmation of product value at the checkout, for example by making ratings and recommendations available, improved the likelihood of impulse purchasing. The vividness and social presence of a website were found to be significant experiential factors in enhancing impulsive purchase motivations. Value perception was then found to be an important factor in converting this purchase motivation into an actual purchase. The study concluded that reaffirmation of product value at the checkout, for example by making ratings and recommendations available, improved the likelihood of impulse purchasing. A shortened payment procedure was also reported to further facilitate an impulsive purchase.

## Emotional Factors

### Theoretical Background: Emotion and Affective States

Emotion and mood are constructs that describe how we are feeling at any given time. Whereas moods are more generic, emotions are more specific. Emotion is typically elicited based on one’s evaluation of the internal or external stimuli (Ellsworth and Scherer, 2003; Pessoa and Adolphs, 2010). The valence of emotion may be positive, neutral or negative depending on the outcome of one’s evaluation. Consumer emotion is linked to attitude (e.g., Batra and Ray, 1986) and preference (e.g., Shiv and Fedorikhin, 1999) and is therefore important throughout all stages of the consumer process in eCommerce. We differentiate between different emotions and mood because the specific remedy for an emotion might differ (see Ellsworth and Scherer, 2003).

It is important to realize that most negative emotions arise when consumer’s goals or expectations are not matched (Moors, 2016). For instance, if the checkout process takes longer than anticipated, if customers cannot add or subtract items at this point, if the description of the items does not allow them to recognize the items and review them at this point, or if additional costs are added consumers will experience such a mismatch of expectation and goals and subsequent irritation or anger (Moors, 2016). All these emotions can result in consumers’ hesitation to continue with shopping process and has sometimes be described as (pre-) decisional conflict (Erdil, 2018; Huang et al., 2018). It is therefore important to reduce any of these uncertainties (Tang and Lin, 2019). In contrast positive emotions often signal that an activity is safe (Schwarz and Clore, 2003), or valuable and achievable (Louro et al., 2007; Fishbach et al., 2010), which causes people to continue with it. Indeed, instilling positive emotion may even promote spontaneous purchase (Verhagen and van Dolen, 2011). For example, good website design has been linked to shopping enjoyment, and subsequently impulsive purchasing behavior in a recent model (Floh and Madlberger, 2013; see also Korzaan, 2003). In what follows, we will describe how negative emotions can prevent transactional success but also how positive emotions can facilitate it. We also discuss how negative emotions can be reduced or even prevented and how positive affective states can be fostered.

#### Customer Irritation and Disappointment

An affective cause of abandonment of an online shopping cart has been posited as customer irritation. Irritation has a negative impact on consumer behavior in traditional retail environments, particularly in women (D’Astous, 2000), and this is likely to translate to online environments. For example, Hasan (2016) observed negative correlations between irritation and navigational and informational aspects of website design. Navigational design was found to have the greatest impact on consumer irritation.

A commercial United Kingdom online survey (Worldpay, 2016) reported that 67% of transactions were abandoned at the checkout stage. Top reasons included having to register for an account, fees for alternative payment methods, and lack of trust in site security (see also Baymard Institute, 2019). Additional factors associated with cart abandonment during the final stage of a transaction include transaction inconvenience, perceived waiting time, and risk (Rajamma et al., 2009). Recent work has also underlined the importance of organization of items within the cart in order to prevent shopping cart abandonment (Xu and Huang, 2015). In particular, the inconvenience of lengthy forms to fill in, delays in processing checkout information, and the heightened perceptions of risk that arise when delays occur often prompt consumers to cancel a transaction. In addition to irritation, mismatches between customer expectation and experience are also likely to promote customer disappointment, thereby further increasing the likelihood of abandonment of the transaction.

As evidenced by metric data from real online transactions, many sources of irritation can be reduced by simple means. For instance: using visual bars to indicate progress through the checkout process, creating realistic expectancies of how long it will take (Sheth, 2013); allowing consumers to change the order without having to re-start the shopping process; and by providing transparency over costs throughout the entire process (e.g., by providing shipping costs prior to the checkout stage, Sheth, 2013). Further, transactions will also be strongly influenced by the fluency and simplicity of the checkout process (Indiani and Fahik, 2020). This can be achieved by one-click or minimal checkout procedures (Dutta et al., 2003; Park and Kim, 2010; Shen and Khalifa, 2012). Importantly, consumers experience more fluent online shopping experience as less effortful and feel more positive toward the experience and the choice made (Mosteller et al., 2014).

It may not be possible to simplify all aspects of the checkout procedure. For example, although some sites remove the need for registering by allowing checkout via external e-payment routes where address and payment information are already stored, for many eCommerce sites it is necessary (and desirable for the company) for new users of the site to register and create a profile. This is a laborious step and one where baskets are frequently abandoned. This is particularly relevant in mCommerce where may be difficult to enter large amounts of information on a mobile device, and/or the requested information may not be easily to hand. Where there are costs to the user (in terms of time and effort), sites may offset this by (a) acknowledging the extra work the user must engage in and (b) providing an immediate benefit/incentive for doing so (e.g., by registering today, we’ll offer you free shipping/a discount). By taking both steps, the retailer can potentially build a reciprocal relationship with the user (Molm et al., 2007), and help reduce the psychological impact of the extra time and effort spent (Rohn, 1998), although these theories currently remain untested in an online retail environment.

#### Security Fears

Privacy and security concerns are paramount in the minds of online shoppers when divulging personal and financial information (Sørebø, 2018). For instance, perceived risk was observed to be a significant predictor of transaction abandonment (Rajamma et al., 2009; El Haddad et al., 2018). Consumers were concerned that the company might misuse their information or that details may be stolen due to poor site security. Consumers were particularly concerned if security features were not evident at the checkout. Fears were heightened if the consumer was not familiar with the company. Indeed, these concerns were observed to supersede concerns over products and services when considered together (Mousavizadeh et al., 2016). However, Mousavizadeh et al. (2016) also showed that assurance statements (statements of policy and procedure relating to privacy and security issued by the vendor) and third party assurance seals (awarded for good business practice by independent bodies) were found to significantly allay many of these consumer fears, as indicated by an increase in purchase intention (see also Özkan et al., 2010).

A more detailed analysis of perceived risk in online shopping (Yang et al., 2015) found that many forms of perceived risk associated with online shopping (functional, social, service, psychological, economic, time, privacy, and security) could be more simply categorized as system risk and transaction risk. System risk, relating to the website payment infrastructure, may be reduced by assurance statements and seals. Transaction risk, relating to transaction parties and processes was found to be unaffected by such assurances. This type of perceived risk was observed to increase with increasing transaction size and is likely to be assuaged by online payment protection against fraud, such as that currently provided by credit cards.

Different factors that impact consumers’ perceived risk have been identified including attitude to risk (Chu and Li, 2008), and differences in decision making styles (Chang and Wu, 2012). Risk reduction strategies such as selecting a product to purchase according to seller’s reputation, brand, endorsement, or recommendation are found to be effective in reducing these perceived risks. Indeed, reputation has been notably associated with lower risk perception for high involvement products (Moore and Mathews, 2006).

The costs to all transaction parties, both financial and experiential, have been highlighted as an important factor in the development of new online payment technologies (Peffers and Ma, 2003). Historically the growth of eCommerce has been hindered by often inappropriate payment systems that were originally developed for offline use. Successful future systems need to incorporate system features appropriate to online retail. Buyer anxiety may be reduced by not having to give credit card information directly to a vendor, and deferred payment and credit schemes may encourage parties without credit cards to shop online, particularly for high cost items. Indeed, possession of an online deferred payment account has been observed to be a positive determinant of the percentage of income spent online (Hannah and Lybecker, 2010).

Importantly, Garbarino and Strahilevitz (2004) found that female shoppers perceived the risks of online shopping to be greater in terms of both likelihood of occurrence and impact. Women perceived the consequences of loss of privacy to be greater than those perceived by men. However, it was found that receiving a website recommendation from a friend substantially mitigated the perceived risk of shopping online in women, as evidenced by an increase in willingness to buy. The same was not found to be true for men.

#### Trust

Risk and security are clearly important factors. Indeed, early online trust researchers predicted that assuagement of consumer concerns over the storage and handling of personal information would be key to maintaining online consumer trust (Hoffman et al., 1999). More than 20 years on, consumer concerns over secondary use of personal information (such as data mining) and hacking fears remain highly relevant. However, it has been observed that trust in an online merchant plays an important mediating role (Lee and Turban, 2001). Familiarity and trust are key for reducing fear and keeping up positive emotions (Gefen, 2000). Antecedents of online trust include perceptions of privacy and service quality, and consequences include satisfaction, loyalty, and repeat purchase intention (Kim and Peterson, 2017). Indeed, Kniberg (2002; in Özkan et al., 2010) reported that customers were more likely to use an insecure payment system if they trusted the company, and conversely, were less likely to use a secure payment system if the company was not trusted. Therefore, the importance of promoting a trustworthy brand should not be underestimated. A study of uptake of online payment options in United States eBay transactions (Black, 2005) found that trust in online payment systems increased according to a number of demographic factors: Internet exposure and experience; Gender (males are more likely to trust online payment systems); Level of education; Income; Geographic location (rural dwellers are less likely to trust online payment systems). Other factors including culture and website type are also known moderators of the online trust relationship (Kim and Peterson, 2017). Currently credit and debit cards remain the most popular mode of online payment, but PayPal and similar services are close behind in popularity (Twenga Solutions, 2016). The adoption and continued use of mPayment technology has been shown to be dependent on personality, beliefs, and social influence; however, social influence exerts the greatest effect at the technology adoption stage (Yang et al., 2012). Barriers to consumer adoption of mPayment technology include cost, risk, and attractiveness of alternatives. Uptake of new payment technology is also dependent on merchant adoption, where barriers include transaction fees, compatibility issues, and a lack of relative advantage (Dahlberg et al., 2008).

Further, trust has been shown to be established through recommendations via word of mouth (e.g., Awad and Ragowsky, 2008). One potential mechanism for this effect may be the reduction of social risk (related to self-esteem and self-confidence) that is negatively associated with consumer trust (Han and Kim, 2017). Therefore, vendors should look to maximize website familiarity via marketing exposure including advertising and word of mouth campaigns.

Interestingly, further study of gender differences (Rodgers and Harris, 2003) found that negative aspects of emotion, trust and convenience were strong predictors of women’s dissatisfaction with online shopping and thereby reflected women’s actual shopping behavior. Indeed, perceived benefits of online shopping have been observed to be greater for men than women (Chen et al., 2015), although this interacts with level of propensity to trust; high trust propensity women showed the greatest future purchase intentions. Regarding employment status, workers have shown a greater trust in online payment and a greater likelihood than students to adopt new technologies such as mobile payment systems (Lu et al., 2011).

Finally, there is evidence to suggest that after sales support is important in maintaining customer satisfaction and brand trust. Both of which are important factors in customer retention. If at the post-purchase stage there is a disconfirmation of pre-purchase expectations then this disparity will give rise to negative emotions which will severely impact on brand loyalty (Cho et al., 2001). Increased satisfaction and likelihood of return custom could potentially be instigated by providing immediate confirmation of an order and good communication throughout the delivery process.

Interestingly, online brand trust, an antecedent of online brand loyalty, has been found to be determined by a different set of factors to offline brand trust. Privacy, security, and information quality are additional predictors of online brand trust, along with brand name, past brand experience, and word-of-mouth communication that are more like offline factors of brand trust (Ha, 2004). Brand experience has been shown to influence brand familiarity and satisfaction and is the main predictor of brand trust (Ha and Perks, 2005). Therefore, increasing the number and variety of positive brand experiences is key to increasing brand trust (Ha and Perks, 2005).

#### Brand Loyalty

Loyalty to a brand may be indirectly related to transactional success. For example, if a branded product is available across multiple websites, consumers may browse to seek the best deal, but are likely to ultimately select the website to complete their purchase based on one or more of the factors discussed here. It may be inferred from models of brand loyalty that consumers are also less likely to abandon a transaction if they have nurtured a loyalty to the website through previous experience, however, this appears yet to be formally tested in the literature. Loyalty is not only based on cognitive factors; emotional factors also play a role such that loyalty may be maintained despite any negative cognitive experiences, and loyalty may also be transferable between offline and online aspects of the brand (Toufaily et al., 2013). A common measure of such website loyalty is repurchase intention.

A framework for building online loyalty is proposed by Grewal et al. (2004): in order to promote website loyalty, it is helpful to make product and information searching as easy and efficient as possible as search costs are an important factor in repurchase intention. Social costs are also important when building brand loyalty and can be aided by providing an online community and feedback system (Wu et al., 2014). Indeed, providing several different brand related experiences such as chatrooms, bulletin boards, and interactive events will increase satisfaction and brand trust (Ha and Perks, 2005). Promoting social norms in this way is also beneficial to the uptake of m-payment technology (Yang et al., 2012). Informing customers of new deliveries or impending *ad hoc* price cuts is likely to increase website loyalty and positive word-of-mouth, particularly in online bargain shoppers (O’Donnell et al., 2016). In general, a loyal customer is likely to demonstrate a high level of trust and confidence in a website, making abandonment of a transaction less likely. Therefore, it is clearly desirable to introduce design measures that promote website loyalty.

## General Discussion: Future Directions for Research

The literature described above indicates a complex interaction between consumers’ motivation, their varying emotional and affective states, and various other factors such as the types of product they are purchasing, and influential aspects of web design. Though the literature already provides much insight into the factors that facilitate or hinder successful buying there are clearly limitations to the current literature that need to be addressed in order to maximize the validity of the conclusions and enable development of a more comprehensive explanatory model. In what follows, we outline suggestions for future research.

### Impact of Motivation and Emotion

A considerable methodological limitation is that although many studies have considered the topic of transaction failure in online shopping, most studies make use of self-reported survey data rather than controlled experiments. Controlled experiments would for instance allow to test the effects of various incentives to make buying more attractive and/or feasible on actual buying behavior in much more direct ways. Such experimental approaches could also take the type of device, the specific product, and individual differences of consumers into account (see also below). Further, it seems important to investigate whether consumers that originally pursued motivations other than buying when visiting an online store will become buyers at a later point. We hope that future research will investigate the factors that turn them into (delayed) buyers. Thus, longitudinal buying studies are needed in order to investigate the motivational drivers for return purchases. Collaboration with online retailers could be one way to gain access to this type of data.

The full range of consumer emotions is also yet to be investigated. For instance, specific emotions could be investigated either by measuring specific emotional profiles of consumers using self-report or by inducing emotions and thus establishing a causal relationship. Importantly, because different emotions are related to different motivations and different ways to alleviate them this would help to highlight emotion-specific strategies (Roseman et al., 1994; Yi and Baumgartner, 2011). Emotions such as shame, guilt, and regret seem highly relevant for future research in order to clarify their impact on shopping cart abandonment and in order to highlight potential ways to alleviate them.

For instance, shame and/or guilt are likely to occur either when anticipated at the purchase stage where they may contribute to shopping cart abandonment, or post-purchase which may result in the immediate return of unwanted products. As another example, complicated check-out procedures and use of technical language might cause consumers to feel shame or embarrassment. This is likely for consumers that feel already uncertain about their lacking technical skills such as older consumers. Those consumers are likely to withdraw in response to these feelings (Lockhart, 2014). Future research could not only test whether shame and embarrassment are indeed evoked in these situations but also test whether messages that reduce feelings of shame by indicating that having trouble is normal (‘Don’t understand what’s going on? No worries, we are here to help’; cf. Leach and Cidam, 2015) are effective in reducing its detrimental effects. In contrast, guilt can be evoked when consumers see the final price of their shopping in the check-out stage which they underestimated. This might cause them to quit the purchase all together because this would reduce or repair feelings of guilt via ‘punishing’ themselves (cf. Nelissen and Zeelenberg, 2009). Future research could test whether checkout guilt is assuaged by providing cost updates throughout the shopping experience or by offering to subtract items at the checkout stage or alternative payment options that delay paying.

Similarly, anticipated regret most likely occurs during checkout because checkout is likely to be the place where consumers first come face to face with the overall costs of their basket. Especially for large overall baskets or single big-ticket items, the scale of the overall cost is likely to act as a trigger for consumers to reconsider their decisions and look at possible alternatives (Zeelenberg et al., 2000). Future research should therefore test whether feelings of regret at checkout could be reduced by offering visible and cost-free return policies, and price guarantees. From a marketing perspective price guarantees and return policies can often be low cost options for the seller. In sum, future research should test the role of specific emotions and their specific remedies in shopping cart abandonment.

### Processing Mode

Our review shows that a wide range of different motivations, emotions and affective states cause or prevent transactional success. A crucial challenge for future research is to understand how these different factors interact in online shopping behavior. We suggest that future research might benefit from applying models that consider differing modes of processing (Petty and Cacioppo, 1986; Kahneman, 2003; Strack and Deutsch, 2004). This will also allow to account for types of product, device, and specific characteristics of consumers.

These theories distinguish two routes by which people think, feel, and behave (Petty and Cacioppo, 1986; Strack and Deutsch, 2004). One is quick, impulsive, and driven by emotions (e.g., spontaneous purchases) whereas the other is slow and based on analytical, logical thought such as carefully comparing and weighting information when making a purchase. The so-called automatic or hot system evolved to react very quickly to rewards or dangers in our environment. In contrast, the slower, more rational system allows us to overcome these impulses and to weight information carefully, often leading to choices that are more beneficial in the long run.

We argue that both systems can drive and prevent a purchase: the automatic, hot system might cause a consumer to quickly buy a tempting product but the hot system might also prevent a purchase when something in the shopping experience causes fear or irritation which will make the consumer abandon the process (cf. LeDoux, 2001). Similarly, the analytical system can stop the consumer from buying a desirable product when it is not in line with the consumers’ saving goals (Hofmann et al., 2008) but it can also promote a purchase of a useful, high quality, or good value item without the product or website being overtly exciting or visually appealing (cf. Lien, 2011). Further, when choice is difficult, consumers often choose to defer a purchase decision, unless they are thinking more analytically (Dhar, 1997; Savary et al., 2015). We suggest that whether a purchase decision is impulsive or more rational depends on the person, the product, and device.

For instance, consumers will be processing in depth if they have time, motivation (e.g., when the purchase is important or expensive), and resources or energy to do so (Petty and Briñol, 2015). If they have little time, are less motivated to process all information, are tired or otherwise missing the resources or energy to think in depth and analyze information (e.g., when using mDevices), they will be more influenced by superficial factors such as the visual appeal of a website or product or the fame of a seller or brand. However, some people may tend to think more analytically and in depth than others (Petty and Briñol, 2015).

Irrespective of any demographic differences, a highly emotional product such as a non-essential fashion item is much more likely to evoke an emotional and automatic thinking mode in the consumer (Hofmann et al., 2008) whereas a utilitarian product is much more likely to cause the consumer to think analytically about it. Indeed, online information for utilitarian products has been shown to be of greater importance to purchase behavior than information provided for hedonic products (Cheema and Papatla, 2010). Further, the way information is presented can activate a certain processing style. For instance, text and detailed information will instigate an analytical thinking style whereas a more picture-based layout will instigate a less analytical thinking style (Meyers-Levy, 1989). This would suggest that mCommerce devices cause a less analytical thinking style because they represent information in simpler, less text-heavy ways. From this perspective, consumers might be more likely to buy ‘hot’ items via mCommerce devices, which could be channeled by promoting respective applications for mCommerce devices (Wang et al., 2015).

Consumers in an automatic processing style are also more likely to be influenced by superficial attributes of a product such as its look, the fame, beauty, likeability, or the seeming credibility or trustworthiness of a person advertising it (Petty and Briñol, 2015). This could explain why website aesthetics have also been observed to influence purchasing behavior through a moderating effect on customer satisfaction (Wang et al., 2011). Interestingly, an interaction between two dimensions of aesthetics, formality (relating to patterns in the presentation of information) and visual appeal, was observed by Wang et al. (2011). High aesthetic formality and high aesthetic appeal were found to promote impulse purchasing behavior in task-free consumers (browsers). However, if customers were task-oriented (i.e., they had to choose a product) then high aesthetics reduced the likelihood of completing a purchase, whereas low aesthetic appeal facilitated purchase completion. It is possible that ‘style over substance’ fears may negatively impact on product satisfaction under these conditions as would be predicted by the theory outlined above. Relatedly, consumers were less likely to complete shopping purchases in mCommerce when they felt ambiguous about the purchase due to security concerns or concerns to make the right choice that were evoked by the limited information available in the mCommerce store designs (Huang et al., 2018). In a related vein, using mCommerce seems to raise both order rate and size but mostly for habitual purchases, thus items that consumers are familiar with and need less information on (Wang et al., 2015).

A similar survey (Liu et al., 2013) confirmed visual appeal of a website as an important factor in instant gratification and impulsivity. It was found that visual experience was enhanced by website ease of use and perceived product availability. The impact of usability on perceived aesthetics was also confirmed experimentally (Tuch et al., 2012) however, this relationship between usability and aesthetics was not reversible. In a similar vein, usability also appears to promote experiences of flow which is defined as a sense of deep involvement that is intrinsically enjoyable and is related to buying (Korzaan, 2003; Hsu et al., 2012; see Hoffman and Novak, 2009 for an overview on research on flow in online consumer behavior). We hope that future research applies the insights of dual process (or systems) models in order to advance our understanding of shopping cart abandonment.

### New Technologies

Future research will also have to account for changing types of technology as much of the existing literature is outdated. For example, personal digital assistants may be advantageous in removing unnecessary work from time poor or less internet savvy consumers. Wearable technology is likely to engender different online shopping attitudes and behaviors through increased convenience. Augmented reality (AR) provides the potential to see what clothing or make-up might look like on, or what a product might look like in your own home and is likely to impart feelings of ownership resulting in greater likelihood of purchase. Research in this field has already begun, with lab studies suggesting that intention to buy is indeed increased by the use of AR, e.g., Poushneh and Vasquez-Parraga (2017). However, more extensive testing in the home is needed. A qualitative study using an AR make-up mobile app in the home found that consumers viewed this as a fun or entertaining activity rather than a purchase tool (Scholz and Duffy, 2018). Therefore, it seems unlikely that shopping cart abandonment would be reduced under such circumstances. However, such observations may be specific to make-up and may not extend to other products such as clothing or furniture. Clearly continued research is needed in the AR arena. While AR acts as an aid to consumer choice, the internet of things (IoT) has the potential to remove human choice from the purchase decision altogether (beyond the initial decision to install such technology) (see Nguyen and Simkin, 2017 for an overview of IoT). As the online environment is further integrated into our everyday non-digital lives, we may face reduced barriers to online purchasing as such behavior becomes more fluent and normal. We are also likely to experience increased competition between these purchase tasks and our other ongoing activities, as purchase choices will increasingly be made via mCommerce whilst on the move or completing other tasks. Advances in payment technology are also likely to influence future online shopping trends. Modern encryption may reduce much current anxiety over online fraud. Increased availability of deferred payment and online credit systems may increase accessibility to online shopping and reduce trust issues and other negative emotions associated with paying in advance for goods not yet received. In sum, future research needs to pay attention to changing technologies and how they facilitate or impair online buying behavior.

### Demographics

Finally, we hope that future research will also continue to study factors underlying transactional success for different demographics. For instance, further research is needed into the factors that impact older adults when purchasing online. In a large, longitudinal, cross-geographical survey of the “Net Generation” (young adults who have grown up with online technology), significant increases in online purchasing behavior have been seen over time (Comegys et al., 2006). However, barriers to technology adoption still remain (Lee and Coughlin, 2015). Although the new wave of older adults will have grown up familiar with internet technology, the impact of aging on factors such as risk and trust will impact on the future online purchasing behavior of this generation (Lian and Yen, 2014; Chakraborty et al., 2016). Disabilities or limitations such as poor eyesight and arthritis may also impact transactional success. Security systems such as CAPTCHA’s or crowded web pages with small text have already been identified as barriers to online shopping for those who would likely benefit most from being able to shop online (Wolters and Aspinall, 2015). Therefore, future web design should encompass the needs of these groups. Gender differences are not typically observed in this older generation but have been observed in younger age groups. For example in Finland, young adult males were observed to make significantly more online purchase decisions than women and this divide was observed to widen over time. However, in the United States no such gender divide was evident, therefore cultural differences are also evident in online shopping behavior. Indeed, espoused cultural and gendered values have been reported to moderate the effects of perceived usefulness and information quality on e-service adoption and satisfaction (Udo et al., 2012). Although both genders have been equally represented in most research, age related factors have not been fully addressed owing to the large number of studies relying on undergraduate student data.

Further, new “net” generations may also have different motivations from previous generations and are likely to experience different emotions. For example, they may be less fearful but may be quicker to show boredom or impatience, meaning that old research may no longer apply. This will pose new challenges for online retailers and will require ongoing research on motivations, emotions, and behavior of target consumer groups.

### Conclusion

Key factors identified in the prediction of online transactional success include not only the consumer’s initial purchasing goals, but a complex interaction of emotional factors that may be influenced, either positively or negatively, by the online environment. Negative emotions such as irritation, disappointment, or fear clearly reduce the likelihood to buy and need to be managed where possible through good website design. Trust and brand loyalty can be actively promoted. Processing modes can similarly be managed through the quantity and type of information provided. By designing online purchase and payment mechanisms that work with our automatic, heuristic processing mode, the easier and more fluent they will become, thereby increasing the likelihood of transactional success. However, continued research is needed to fully understand the impact that new technology will have on future online shopping behavior.

## Author Contributions

All authors listed have made a substantial, direct and intellectual contribution to the work, and approved it for publication.

## Conflict of Interest

The authors declare that the research was conducted in the absence of any commercial or financial relationships that could be construed as a potential conflict of interest.
